# Temporal Characteristics of Online Syntactic Sentence Planning: An Event-Related Potential Study

**DOI:** 10.1371/journal.pone.0082884

**Published:** 2013-12-20

**Authors:** Inge Timmers, Francesco Gentile, M. Estela Rubio-Gozalbo, Bernadette M. Jansma

**Affiliations:** 1 Department of Cognitive Neuroscience, Faculty of Psychology and Neuroscience, Maastricht University, Maastricht, The Netherlands; 2 Department of Paediatrics, Maastricht University Medical Centre, Maastricht, The Netherlands; 3 Institute of Research in Psychology, Institute of Neuroscience, Université Catholique de Louvain, Louvain-la-Neuve, Belgium; 4 Maastricht Brain Imaging Center (M-BIC), Maastricht, The Netherlands; 5 Laboratory of Genetic Metabolic Diseases, Maastricht University Medical Centre, Maastricht, The Netherlands; University of Leicester, United Kingdom

## Abstract

During sentence production, linguistic information (semantics, syntax, phonology) of words is retrieved and assembled into a meaningful utterance. There is still debate on how we assemble single words into more complex syntactic structures such as noun phrases or sentences. In the present study, event-related potentials (ERPs) were used to investigate the time course of syntactic planning. Thirty-three volunteers described visually animated scenes using naming formats varying in syntactic complexity: from simple words (‘W’, e.g., “*triangle”, “red”, “square”, “green”, “to fly towards”*), to noun phrases (‘NP’, e.g., *“the red triangle”, “the green square”, “to fly towards”*), to a sentence (‘S’, e.g., *“The red triangle flies towards the green square.”*). Behaviourally, we observed an increase in errors and corrections with increasing syntactic complexity, indicating a successful experimental manipulation. In the ERPs following scene onset, syntactic complexity variations were found in a P300-like component (‘S’/‘NP’>‘W’) and a fronto-central negativity (linear increase with syntactic complexity). In addition, the scene could display two actions - unpredictable for the participant, as the disambiguation occurred only later in the animation. Time-locked to the moment of visual disambiguation of the action and thus the verb, we observed another P300 component (‘S’>‘NP’/‘W’). The data show for the first time evidence of sensitivity to syntactic planning within the P300 time window, time-locked to visual events critical of syntactic planning. We discuss the findings in the light of current syntactic planning views.

## Introduction

Language is an important basis for communications with others. As a speaker, we are constantly constructing streams of thoughts and planning messages to transfer these thoughts into the outside world. As a listener, we receive acoustic, visual and contextual information, and integrate this into a meaningful message. Whereas speech production and comprehension (or encoding and decoding) have been separate fields in psycholinguistics, recent discussions argue that they are interwoven, non-isolated processes, that largely share underlying mechanisms (see e.g., [1], [2]). Although a lot is already known about online syntactic processing during comprehension based on electroencephalography (EEG) and functional magnetic resonance imaging (fMRI), comparably less is known for the production analogue. A balanced knowledge is necessary to investigate potential commonalities of syntactic processing in both modalities. The current study focuses on syntactic planning during production and addresses the question when in time syntactic planning for speaking takes place.

There are many accounts on how we apply grammatical rules to be able to generate meaningful utterances. In general, most researchers agree that speaking involves conceptual, syntactic, and phonological planning that leads to articulation. Views differ on whether we should see these processes as serial stages, unfolding over time, or more as parallel processes. In classic serial accounts, speakers carry out syntactic sentence planning in several steps. First, lexical concepts and corresponding syntactic information (e.g., whether it is a noun or adjective; lexical selection) are identified and activated. Secondly, syntactic relations and functions are assigned to each word (e.g., subject versus object; function assignment) and proper inflections are added (e.g., -*s* for plural, -*ed* for past tense). Finally, words are assembled into so called syntactic structural frames (constituent assembly) [3], [4]. Friederici [5], [6] also assumes serial processing, but suggests that syntactic processes first build a local structure, after which grammatical and semantic relations are assigned in a utterance. In an interactive view, Kempen ([2], but see also e.g., [7], [8]) describes a localist neural network model in which grammatical encoding is a task assigned to the Unification space (or U-space). Via a recursive transition network (RTN), activation spreads across so-called treelets or syntagma’s that can be bound to lemmas. A list of annotated lemmas is eventually converted to a list of word forms. The author notes, however, that although processes (conceptual, syntactic) are initiated in parallel, the behaviour of the network may seem serial because some processes may require more time. The stage-like behaviour is therefore only an emergent property of the model. Other views do not assume that a formal grammar (rules) interacts with a mental lexicon (words). They rather consider language as an emergent property, emphasizing the role of the user’s experience [9], [10], [11], [12], [13], [14]. The role of experience, however, is also evident in other, more classic views (e.g., the recursiveness of network models, [2]).

Most theories envision speech production as an incremental process, although the units of increment differ between views [4], [15], [16], [17], but might also vary across speakers (e.g., cognitive capacity, experience), and could be dependent on the situational context (e.g., time pressure) [4], [18], [19], [20]. Further, sentence planning can be either lexically or structurally incremental (one can guide the other, [21]), or a flexible interaction between both. Evidence from a recent study points towards structural incrementality [22], implying a role for structural assembly in early sentence production (i.e., preceding lexical retrieval; and in contrast to psycholinguistic views in which lexical retrieval occurs prior to syntactic planning; [3], [15]).

Whereas there is still the ongoing debate about the exact nature of syntactic planning, only recently studies have ventured to investigate the neural aspects of information processing during the production of complete sentences. Several brain areas have been reported to be involved in syntactic encoding, including the left inferior frontal gyrus (LIFG; BA 44/45/47), left posterior medial temporal gyrus (lpMTG, BA 21), and bilateral supplementary motor areas (SMA; BA 6) [23], [24], [25], [26], [27], [28], [29]. Not much is known, however, about the time course of syntactic encoding (see also [30]). A method of choice to investigate temporal characteristics of information access is EEG, and its derivative, the event-related potential (ERP). For single word production, the experimentally elicited lateralized readiness potential (LRP) [31], [32], [33], and the N200 go-no go component [34], [35], [36] have been extensively studied in single word and noun phrase production. Based on the LRP and the N200 go-no go results, it has been estimated that semantic access precedes syntactic access by approximately 90 ms, which is followed by phonological encoding after around 40 ms, suggesting incremental planning (but also see the discussion in [37]). So far, the most direct measure of the time course of syntactic encoding was carried out via invasive intracranial electrophysiology (ICE). Sahin et al. [38] used ICE to record local field potentials (LFPs) near Broca’s area in patients who had to either read or inflect a word (past/presence or singular/plural). The recordings revealed a component around 320 ms after target word presentation sensitive to (morpho)syntactic processing. In an ERP study, Marek et al. [39] asked participants to overtly describe a walk through a 2D grid consisting of geometric colour figures either in a simple (“go up, go right”), medium (“go up to the circle”), or complex (“go up to the green circle”) manner. They found a P300-like component at 350–500 ms post stimulus onset, distributed over centro-parietal electrodes, that was more positive for medium and complex utterance conditions compared to the simple condition. The authors concluded that the P300 is sensitive to conceptual and/or syntactic complexity variations.

In summary, electrophysiological studies suggest that syntactic encoding is carried out around 300–500 ms after stimulus onset. However, this conclusion is based on indirect measures (LRP/N200 go-no go paradigms), rather than direct naming; based on rather artificial naming tasks (explicit inflection of a certain word within sentence context - which we normally do not do in an highly automatic process); or based on ambiguous interpretations of the data (i.e., no clear separation of conceptualisation and syntactic complexity in the experimental design). In the present study, we used a more direct and natural approach, in order to gain insights into the electrophysiological correlates of syntactic planning. In analogy to a positron emission tomography (PET) study by Indefrey et al. [24], [25], we employed a paradigm where visually animated scenes elicited overt multi-word utterances in a relatively natural way. Participants were instructed to describe the scenes as fast and accurate as possible using a sentence -, a noun phrase -, or a single word format (in Dutch). For example, in one of the visual stimulations a red triangle bumps into a green square. In the *complex, sentence-level* (‘S’) syntax condition participants would describe the scene as “*De rode driehoek botst tegen het groene vierkant op.*” [“The red triangle bumps into the green square.”], in the *medium, noun phrase level syntax* (‘NP’) condition they would illustrate the trial as “*de rode driehoek*”, “*het groene vierkant*”, “*tegen op botsen*” [“the red triangle”, “the green square”, “to bump into”]. In the *minimal syntax, words* (‘W’) condition the correct response would be “*driehoek*”, “*rood*”, “*vierkant*”, “*groen*”, “*tegen op botsen*” [“triangle”, “red”, “square”, “green”, “to bump into”]. The participants were instructed on the type of naming format at the beginning of each block. Visual stimulation was kept constant across conditions.

The rationale of Indefrey et al. behind the three different utterance types was that the required syntactic processing parametrically varied in complexity [24], [25]. Overall the task requires a range of cognitive information processing. The visual scene - identical across conditions - triggers visual and conceptual encoding of motion, colour, and form, as well as of the action (either ‘to fly towards’ or ‘to bump into’). In addition, concepts (i.e., the different geometrical figures plus the verb) must be ordered for serial articulation. Linguistic encoding, depending on the utterance instruction, should trigger the build-up of the appropriate syntactic structure and the filling in of the structure with suitable elements. Following the logic of Indefrey et al., we assumed that the ‘W’ condition required lexical selection of words but virtually no syntactic encoding. In the ‘NP’ condition, syntactic processing was necessary on a noun phrase level, because the retrieval of certain syntactic information and inflections was required (i.e., the article of a noun, inflection of the adjectives, assembly into a phrase). In the ‘S’ condition, syntactic planning was necessary on a sentence level, which includes the processing required in the words ‘W’ and noun phrase ‘NP’ conditions, but also the combination of two noun phrases by adding the verb in its proper form.

The application of high resolution EEG allowed us to time-lock the ERP to certain events within the utterance planning process. We specify two critical events. One event is the scene onset, as it starts the planning of the first elements of the utterance. A second critical event is the moment at which the target action is disambiguated (both scene variations started identical and diverged only from that point on). At that moment, one of the two actions were displayed - unpredictable for the participant. The disambiguating visual moment allowed the speaker access to the target action concept and its syntactic realisation. It also allowed to bind the first noun phrase to the second noun phrase, using the target verb.

ERPs were recorded from the scene onset on. We took a rather explorative approach in this study. Based on a more modular, serial account, we expected that components sensitive to syntactic processing would show a parametric amplitude modulation related to the syntactic complexity variation within a certain time window (based on the additive factor logic [40]). The detected time windows of the parametric modulation should give insights into the time course of syntactic planning stages. Based on the limited electrophysiological literature available [38], [39], we expected to observe a variation with syntactic complexity around 300–500 ms time window after stimulus onset in correspondence of a P300 component, associated with phrase-level syntactic planning. For post-verb disambiguation sentence planning, we aimed to present first empirical evidence with this experiment. In addition, from a more integrative theoretical view, we did not rule out immediate and parallel integration that would affect neural processing in a non-additive manner. This parallel processing might result in early effects in the ERP (i.e., in time windows sensitive to visual and conceptual encoding, [41], [42], [43]). We will discuss the results in the light of the different language accounts.

## Materials and Methods

### Ethics Statement

The ethical committee of the Faculty of Psychology and Neuroscience (Maastricht University) gave clearance for the study. All participants gave written informed consent.

### Participants

Thirty-four healthy volunteers participated in this study. Data of one participant were excluded from the analysis because of the health history and current medication use. Twenty-one of the 33 remaining participants were female. One was left-handed. The mean age was 21.8 years (SD 2.6 years). All had normal or corrected to normal vision and were native Dutch speakers. The participants received financial compensation or received academic credit points.

### Stimuli

Visually animated scenes were presented to the participants. Each scene consisted of three geometrical shapes (square, triangle, or circle) having one of three different colours (red, blue, and green). The individual figures covered approximately 1.6° (height) of visual angle and were configured around the centre (one above and two below the centre on either side). The total configuration covered approximately 5.8° (width)×5.4° (height) of visual angle. In each trial, one of the three geometrical figures performed an action upon another figure: it could either be ‘to fly towards’ or ‘to bump into’. The two scene types started visually identical until they diverged at a certain point (see the *Procedure* section for details on how the scenes differed). In each scene two of the objects could be distinguished by their colour only. This made it more natural to name the colour together with the shape of the objects. The content of the scene was randomly varied across trials (i.e., the shapes, colours, positioning of the figures, and the action). Such variation of the events in the scene was included to keep participants alert and to have online utterance planning on a trial by trial basis.

The paradigm was designed using Presentation 14.0 software (Neurobehavioral Systems, Inc.).

### Procedure

Participants were instructed to overtly describe the presented animated scenes using one of three possible responses: word-‘W’, noun phrase-‘NP’, or sentence-‘S’ format (an example for each condition would be as follows: word-‘W’ - *“driehoek”, “rood”, “vierkant”, “groen”, “naar toe vliegen”* [“triangle”, “red”, “square”, “green”, “to fly towards”]; noun phrase-‘NP’ - *“de rode driehoek”, “het groene vierkant”, “naar toe vliegen”* [“the red triangle”, “the green square”, “to fly towards”], and sentence-‘S’ - “*De rode driehoek vliegt naar het groene vierkant toe.”* [“The red triangle flies towards the green square.”]) (see the *Introduction* for an example of the ‘to bump into’ scene types). After having received instructions, a practice version consisting of 3 blocks (i.e., one per condition) containing 18 trials each was started. The practice session was followed by the main experiment, which consisted of three runs. A single run consisted of three blocks (one per naming condition). The order of naming conditions was randomized within each run (i.e., six possible run types) and across participants.

**Figure 1:**
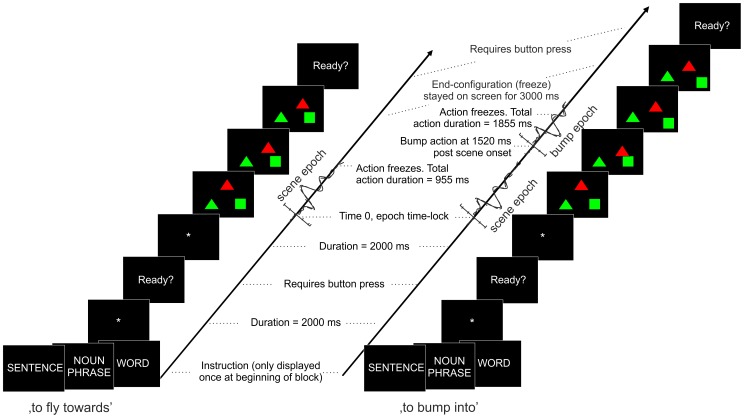
Overview of trials. Schematic overview of the experimental trials, separately for the two action formats (‘to fly towards’ and ‘to bump into’). For illustrative purposes, only screenshots of the trials are displayed (the objects were actually moving). The displayed ERP epochs illustrate the different time windows of interest for the analysis (scene epoch, immediately starting after scene onset; and bump epoch, after the ‘bump’ event and hence after disambiguation of the target verb). Note that action formats are randomized across trials and are not instructed nor predictable to the participants.

Each block started with a brief instruction reflecting the type of naming format to be performed (i.e., either ‘SENTENCE’, ‘NOUN PHRASE’, or ‘WORD’), followed by 40 trials, consisting of a different scene each (see *Stimuli*). A total of 120 trials were recorded for each condition. Each trial started with a fixation point (white asterisk on a black background) for 2000 ms, followed by the display of the geometric figures that moved. The duration of animation in the scene differed (955 or 1885 ms), depending on the action format (‘to fly towards’ or ‘to bump into’, respectively). The difference in animation durations was due to a different amount of action frames (10 versus 18 frames, where the actual ‘bump’ event occurred at frame 14, at 1520 ms after scene onset). The two scenes types associated with the two different actions were visually identical until the moment that the ‘to fly towards’ trials froze while ‘to bump into’ trials continued. The stimulation always ended with a freeze configuration lasting 3000 ms (see Figure 1). Participants were instructed to start the description of the scene as fast and as accurate as possible, and to minimize eye movements. The next trial started via a self-paced button push (by USB keyboard key). This self-pacing format was chosen to take into account inter-individual differences in naming onset and duration. An entire trial took approximately 8000 ms (fixation, scene, freeze time and button to switch to the next trial to continue).

During the recordings, participants were seated in an electrically-shielded, sound-attenuated room in front of a computer monitor (distance approximately 80 cm).

### Apparatus and EEG Recording

The EEG was measured using an elastic cap in which 32 tin electrodes were mounted (Electro-Cap International (ECI), Inc.), positioned according to the international 10–20 system [44]. The signal was recorded from twenty electrodes - F3, Fz, F4, FC3, FCz, FC4, C3, Cz, C4, CP3, CPz, CP4, P3, Pz, P4, O1, Oz, O2, T3 and T4 - referenced online to the left mastoid (A1). Offline the signal was re-referenced to the average signal of both A1 and A2. Vertical eye movements and blinks were monitored by two electrodes placed at the left upper and the lower orbital ridge. Horizontal eye movements were recorded with electrodes placed on the left and right cantus. The impedance of all electrodes was kept below 5 kΩ. Data acquisition was done using Brain Vision Recorder software (Brain Vision, MedCaT B.V.) and the signal was amplified using a 0.05–50 Hz band pass and sampled at a 2 ms-interval (500 Hz). The scene onset as well as the voice onset triggered a code pulse. The code was written directly into the EEG recordings and was used for later event-related analyses. The voice onset was recorded via the microphone and transferred as onset time pulse into the log file. The pulse was triggered when the sound pressure level reached a certain threshold (individually adjusted for each participant).

### Analyses

For the behavioural data, the number of errors (i.e., any deviation from the expected utterance: incorrect object, colour, action, naming format, or ordering) and corrections (i.e., any overt corrective effort during the response utterance) were computed using the recorded audio data and the manual scores collected online by the experimenter. Dysfluent speech was not necessarily coded as a error or correction, only if there was overt corrective effort or a mistake. Moreover, we computed the voice onset time (VOT) as the time between the onset of the scene and the onset of the overt naming. We also calculated the total speech time (TST) that was defined as the time difference between naming onset and the button response. VOTs<0.5 seconds and >4.5 seconds and TSTs<2 seconds and >10 seconds were considered outliers and were discarded from the analysis. A repeated-measures General Linear Model (GLM) with syntactic complexity as factor (3 levels: W, NP, S) was used to analyse the behavioural data.

With respect to the EEG data, trials in which the participant’s response was incorrect, corrected or absent were excluded from further analysis. The EEG data related to the correct naming trials were epoched from −200 to 2500 ms post stimulus onset (to include the entire interval from the onset of visual scene to the end of the display/onset of articulation), band pass filtered from.3–30 Hz (zero phase, 24 dB), and baseline corrected (from −200 to 0 ms).

In order to reduce artefacts in the signal, Independent Component Analysis (ICA) was used. ICA blindly decomposes the multi-channel EEG data into temporally maximally independent components (which computationally corresponds to components sharing the least mutual information) [45], [46]. An Independent component (IC) is characterized by a time course and a scalp topography reflecting the contribution (weight) of that component to the EEG signal at each of the scalp channels (not to be confused with traditional ERP scalp topographies). The ICs typically consist of brain or non-brain (artefact) processes, or are comprised of noisy data (e.g., large, atypical movements do not share mutual information with the other sources and hence would fall into separate unreliable ICs). Non-brain artefact-related (e.g., stereotyped eye blinks, eye movements, and muscle movements) and noisy ICs can be identified by visually inspecting the corresponding topographies and time courses. By removing such ICs, one can filter out the contributions of those processes to the signal. Therefore, this procedure allows de-noising the data, without losing trials and hence statistical power.

In our procedure, we first removed (in the original EEG space) the large and atypical artefacts from the data based on visual inspection to avoid that ICA would extract unreliable ICs devoted to noisy data. On average, 82.1% of the trials were kept for further analysis: 97 trials in ‘S’, 98 in ‘NP’ and 102 in ‘W’ condition. Then, the data were decomposed using the infomax algorithm in EEGlab ([47], http://www.sccn.ucsd.edu/eeglab). Scalp map topographies and time courses associated with all ICs were used to identify those components related to stereotyped artefacts which were removed from the data (e.g., eye movements and blinks typically show a far-frontal projection on the map, and are easily spotted by inspecting the time course; muscle artefact components have a typical spatial localization to the temporal sites and show high power at the high frequencies). This was done individually for all participants (on average 7.8 component per dataset, corresponding to 31% of components).

The remaining, task-relevant components were back-projected onto the original ERP data space and were averaged across trials, separately for each condition. In the back-projected ERPs, epochs were divided in two time ranges: one time interval was time-locked to the onset of the scene (preceding the ‘bump’ event; −200 to 1000 ms after onset of the scene), and one was time-locked to the ‘bump’ event (−200 to 800 ms after the ‘bump’ event, or 1320 to 2320 ms post scene onset; see also Figure 1). Only the ‘bump’ event was considered in the further analysis, because it is a visual event to which the data can be time-locked (such an event is absent in the ‘to fly towards’ trials). Most importantly, from this time on, it was definite which of the two action verbs applied (‘to bump into’ or ‘to fly towards’). Prior to this point, the speaker could still doubt on which of the two events were to be described, and hence he or she could not anticipate and prepare a description of the event (chance level). Note that in the ‘bump’ epoch, less trials were included (only the ones in which the figures bumped and not the ones in which the figures flew towards each other, as the visual stimulation differed between these), corresponding to on average 46 trials in ‘S’, 47 in ‘NP’ and 49 in ‘W’. For three participants, information on the scene types was not available, hence the analyses on the ‘bump’ epoch were performed on the remaining thirty participants. The ‘bump’ epochs were baseline corrected (−200 to 0 ms after the ‘bump’ event).

Based on visual inspection of the grand averages (averaged across all participants), target ERP components and corresponding time windows were specified. Time windows were chosen around the component’s maxima (i.e., either in a standard way [peak latency plus and minus 30 ms for instance] or – especially for later, more variable, components – relying on the data itself to choose the most appropriate range) and were kept constant across conditions. For each ERP component, the mean signal per condition and participant was computed.

Statistics on ERP data were performed on the mean amplitude data (computed per time window, per condition, and per participant). We used a repeated measures General Linear Model (GLM) with syntactic complexity as within-subjects factor (3 levels: W, NP, S) together with two topographical factors: laterality (left, central, right) and anterior-posterior (F, FC, C, CP, P, O) (i.e., in the omnibus tests, a combined total of 18 electrodes were included). Main effects and interaction effects were inspected. Based on interactions between topographical factors and condition, additional analyses were performed on subsets of electrodes. In case of main effects, linear contrasts were inspected first. In case the linear contrasts did not describe the data well, pair-wise comparisons were inspected. Corrections for multiple testing (Bonferroni, in case of pair-wise comparisons) and for sphericity violations (Greenhouse Geisser) were made when necessary. Extreme outlier values (>3*interquartile range) were excluded from the analysis. An alpha of 0.05 (corrected) was used as significance level.

## Results

### Behavioural Data

#### Accuracy

**Figure 2:**
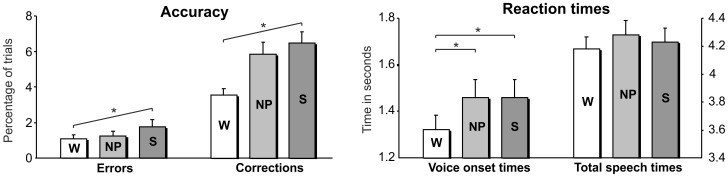
Behavioural data. Mean accuracy (left panel) and reaction times (right panel) per condition. Reaction times (plus standard errors) are displayed for voice onset times (VOT; left axis) and for total speech times (TST; right axis). Asterisks indicate significant linear trends (observed for accuracy) or significant contrasts (observed for VOT). ‘W’ = minimal syntax, word condition, ‘NP’ = noun phrase-level syntax condition, ‘S’ = sentence-level syntax condition.

The number of errors varied linearly with syntactic complexity: the more complex the syntax, the higher the number of errors (linear contrast: *F*_1, 32_ = 7.42, *p* = .010; main effect of syntactic complexity: *F*_1.66, 53.04_ = 3.83, *p* = .035; ‘W’: mean 1.09%, SE.23%; ‘NP’: mean 1.29%, SE.25%; ‘S’: mean 1.81%, SE.38%). The same effect was observed for the amount of corrections (linear contrast: *F*_1, 32_ = 19.56, *p*<.001); main effect of syntactic complexity: *F*_1.84, 58.83_ = 14.00, *p*<001; ‘W’: mean 3.50%, SE.38%; ‘NP’: mean 5.86%, SE.69%; ‘S’: mean 6.48%, SE.68%; see Figure 2).

Inspecting any potential differences in accuracy measures between the two action verbs revealed no main effects of action verb, nor any interaction effects (errors: main effect of action verb *p = *.116, interaction effect *p = *.351; corrections: main effect of action verb *p* = .276, interaction effect *p* = .157).

#### Reaction times

The voice onset times (VOT) revealed a main effect of syntactic complexity (F_1.46, 40.81_ = 20.00, *p*<.001). Contrast analyses showed that in the ‘W’ condition the latencies were significantly shorter compared to both the ‘NP’ and ‘S’ condition (*P*<.001, for both cases; ‘W’: mean 1.30 s, SE.061 s; ‘NP’: mean 1.43 s, SE.073 s; ‘S’: mean 1.43 s, SE.073 s). Analysis of the total speech time (TST) revealed a main effect of syntactic complexity (*F*_1.52, 42.53_ = 4.65, *p* = .023; ‘W’: mean 4.21 s, SE.083 s; ‘NP’: mean 4.32 s, SE.098 s; ‘S’: mean 4.28 s, SE.092 s), but the contrast analysis failed to find any significant differences (Figure 2).

Analysis of the action verbs revealed no main effect and no interaction effect for the VOTs (main effect *p* = .085, interaction effect *p* = .884). However, there was an interaction between action verb and condition in the TST (*p* = .017). Follow up analysis showed that the ‘to bump into’ trials resulted in higher TST in all conditions (all *p*<.001; on average 4.50 s versus 3.96 s). Further, only in the ‘to fly towards’ trials, there was a condition effect (*p* = .010): TST was highest for the ‘NP’ condition. ‘NP’ differed significantly from ‘W’ (*p*-*corrected* = 0.03) and marginally significant from ‘S’ (*p-corrected* = .069).

### ERP Data

**Figure 3:**
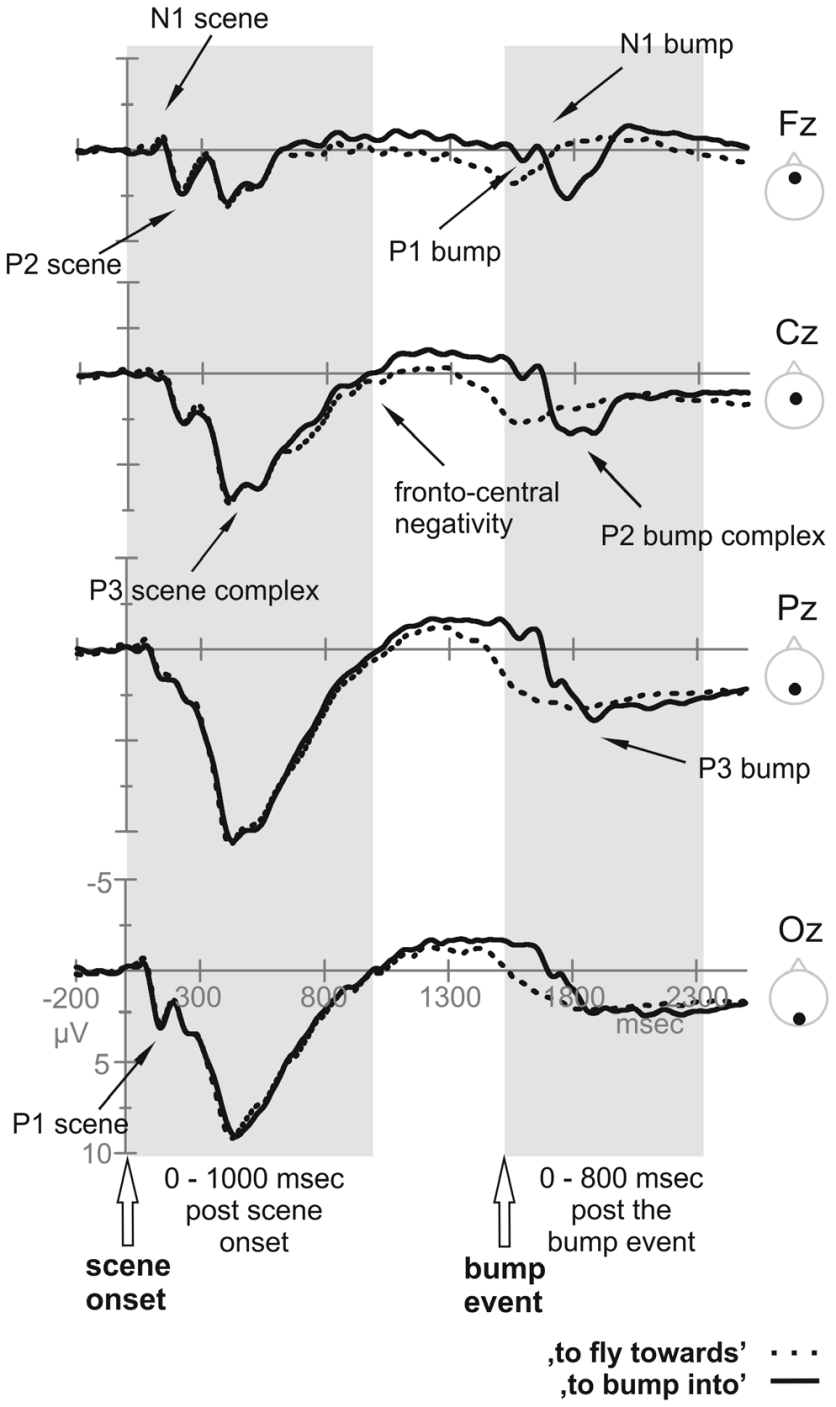
Overview of ERPs. Grand average ERPs, separately for the two action formats (solid lines = ‘to bump into’; dashed lines = ‘to fly towards’), across the midline of the scalp (F = Frontal, C = Central, P = Parietal, O = Occipital) for the entire epoch interval of −200 to 2500 ms after scene onset, reflecting the speech planning from stimulus onset onwards. The two time windows of interest are highlighted: the post scene onset time window (where scenes of both action formats, and their corresponding ERPs, are still identical) and the post bump event time window (where the analysis was limited to the ‘to bump into’ trials, as the ‘to fly towards’ trials did not show an ERP morphology during this time window). Target components are indicated by arrows. Negative voltage is plotted upward in this and all subsequent figures. Note that for plotting purposes, ERP waveforms underwent a low pass filter (5 Hz, 6 dB cut-off).

Visual inspection of the grand averages showed a clear ERP morphology during the first 1000 ms post scene onset, followed by a relatively steady period (in which no event-related activity was visible) (see Figure 3). Another subset of ERP components was observable at a relatively late time interval (from approximately 1500 ms after scene onset onwards), in correspondence of the ‘bump’ event when the target verb was disambiguated (i.e., ‘to bump into’ instead of ‘to fly towards’). The statistical analysis was focused on these two epochs of interest: the first ranged from −200 to 1000 ms after the scene onset and prior to the ‘bump’ event (before the action format and thus the verb was available) and the second was related to the time window between −200 to 800 ms after the ‘bump’ event (when the verb was available, corresponding to 1320 to 2320 ms after scene onset, limited to the ‘bump’ trials). Statistics were carried out across several time windows. Components belonging to the −200 to 1000 ms post scene onset time-window were labelled as ‘scene’ components. These were the *P1 scene* (90–150 ms), the *N1/P2 scene* (100–240 ms), the *P3 scene* (350–550 ms), and the *fronto-central negativity, post scene* (600–900 ms). The components following the ‘bump’ event were defined as ‘bump’ components, namely the *P1/N1 bump* (20–150 ms), *P2 bump* (140–280 ms) and the *P3 bump* (300–500 ms) (Figure 3). Note that the labels P1, N1, P2 and P3 are used for descriptive purposes. P1 refers, for instance, to the first positive voltage inflection, N1 to the first negative voltage inflection and so forth.

#### Time windows of interest post scene onset

*Time window 90–150 ms – P1 post scene:* A positive deflection was observed in the 90–150 time window with a clear occipital distribution and a peak around 120 ms post scene onset. Within this time window, no syntactic complexity effects (*F*_2.00, 63.91_ = 0.187, *p* = .830), nor any condition-related interaction effects (*p*>.15) were found.

*Time window 100–240 ms – N1/P2 post scene:* In the 100–240 ms time window, a negative-positive complex was observed with two frontally distributed maxima: a (rather small) negative component peaking at 130 ms post stimulus, followed by a positive component with a maximum around 210 ms post stimulus onset. The N1 was analyzed in the 100–160 ms post stimulus onset window, and showed no effects of syntactic complexity (*F*_1.98, 63.45_ = 0.054, *p* = .946), nor any syntactic complexity-related interaction effects (*p*>.19). In the P2 time window (180–240 ms post stimulus), also no syntactic complexity effects (*F*_1.92, 63.43_ = 1.616, *p* = .208) and no interaction effects (*p*>.17) were found.

*Time window 350–550 ms – P3 post scene:* During the 350–550 ms time-window, a positivity consisting of a parietal and a more anterior distributed component was observed. The parietal distributed positivity evolved between 350 and 450 ms with a maximum around 390 ms after stimulus onset. The analysis revealed no syntactic complexity effect (*F*_1.99, 63.82_ = 0.080, *p* = .923). There was a significant interaction effect between syntactic complexity and the anterior-posterior factor (*F*_2.93, 93.86_ = 4.178, *p* = .008), but follow up analyses revealed no significant effects per anterior-posterior plane (*p*>.15).

**Figure 4:**
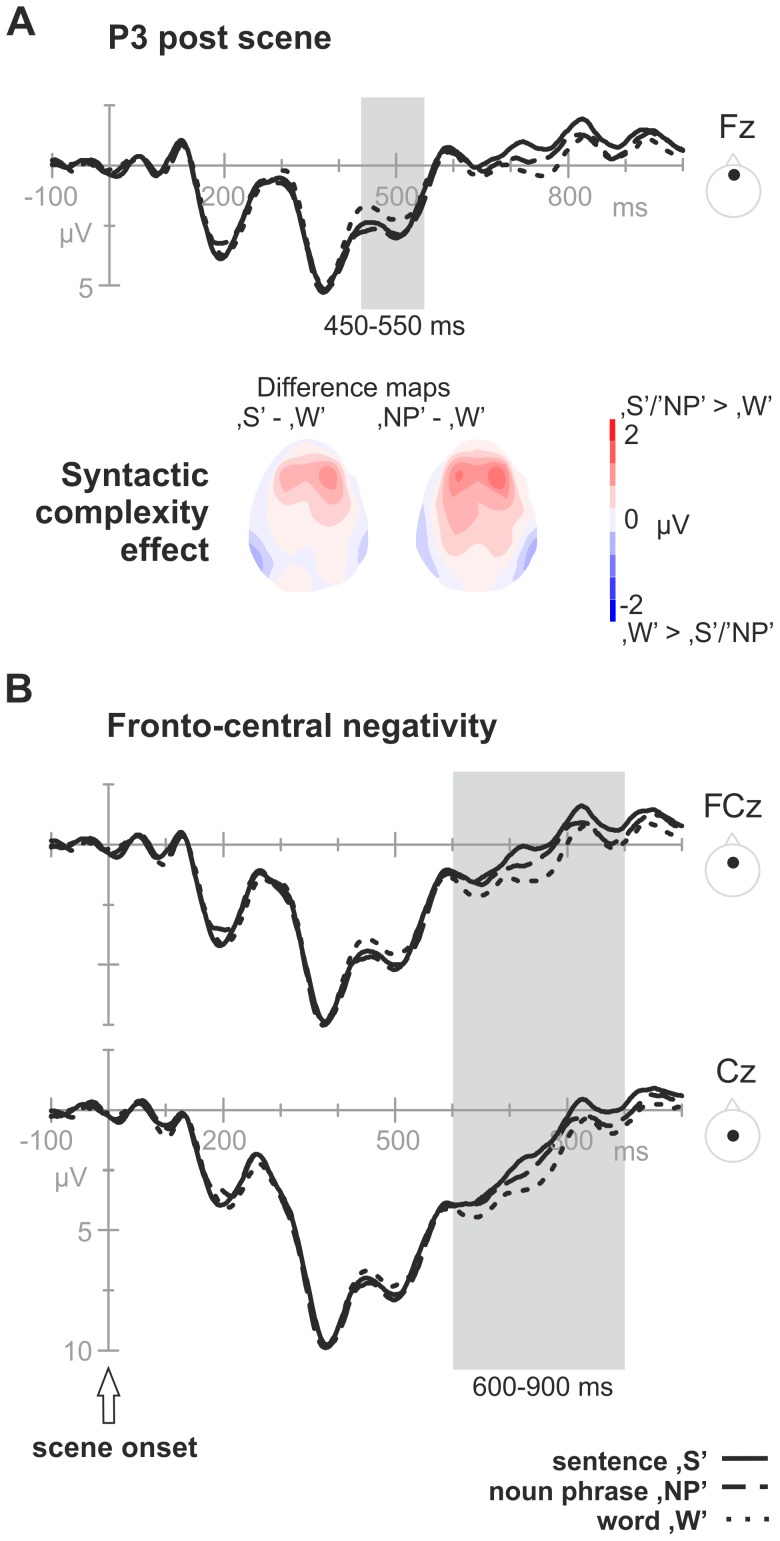
Syntactic complexity effects following scene onset. Grand average ERPs, separately for the three syntactic complexity formats (‘S’, ‘NP’, ‘W’), in the post scene onset epoch. A) The P3 syntactic complexity effect (‘S’/’NP’>‘W’) at frontal midline electrode (Fz) within the time window 450–550 ms post scene onset, together with the topography maps of the effect distribution across the scalp (bottom; left: ‘S’ minus ‘W’; right: ‘NP’ minus ‘W’). B) The fronto-central negativity that modulated with syntactic complexity (linear effect: ‘S’>‘NP’>‘W’) at fronto-central and central midline electrodes (FCz, Cz) within the time window 600–900 ms post stimulus onset.

The second component was analyzed in the time window 450–550 ms post stimulus onset. The overall analysis revealed a trend towards a significant interaction between syntactic complexity and anterior-posterior (*F*_2.59, 82.88_ = 2.643, *p* = .063). Simple contrasts showed that only at frontal electrodes (F), a significant syntactic complexity effect was present (*F*_1.91, 63.10_ = 3.99, *p* = .025) (FC: *p* = .287; C: *p* = .719; CP: *p = *.772; P: *p* = .976; O: *p* = .801). Pair-wise comparisons at F showed a significant difference between ‘W’ and ‘NP’ (*F*_1, 32_ = 6.31, *p-corrected* = .017) and a trend towards a difference between ‘W’ and ‘S’ (*F*_1, 32_ = 4.00, *p-corrected* = .054) (see Figure 4A).

*Time window 600–900 ms – fronto-central negativity post scene:* A negative component was most prominently visible at fronto-central sites in a rather late time-window (600–900 ms post scene onset). In the overall analysis, a trend towards an interaction effect was found between syntactic complexity and anterior-posterior (*F*_2.59, 83.02_ = 1.83, *p* = .055). Simple effect analyses on the fronto-central plane (FC, C) revealed a significant syntactic complexity effect (*F*_1.98, 63.22_ = 3.60, *p* = .034) (at other electrode planes, *p*>.05). Contrast analysis confirmed a linear relation (*F*_1, 32_ = 6.80, *p* = .014): higher syntactic complexity related to higher negativity of the target amplitude (see Figure 4B).

#### Time windows of interest post bump event

*Time window 20–150 ms – P1/N1 post bump:* In the 20–150 ms time window, a positive component was observed (20–80 ms post bump event) followed by a negative component (70–150 ms post bump event), both having a central distribution. No significant syntactic complexity effects were found (P1 component: *F*_1.63, 47.32_ = 2.053, *p* = .148; N1 component: (*F*_1.80, 52.07_ = 2.928, *p* = .068), nor any significant interactions effect in either component (all *p*>.1).

*Time window 140–280 ms – P2 post bump:* In this time window, a component complex was visible with an earlier posterior distribution and a later fronto-central topography. Within this 140–280 ms time window, no significant syntactic complexity effect was found (*F*_1.64, 47.50_ = 2.64, *p* = .091), nor any condition related interaction effect (*p*>.17).

**Figure 5:**
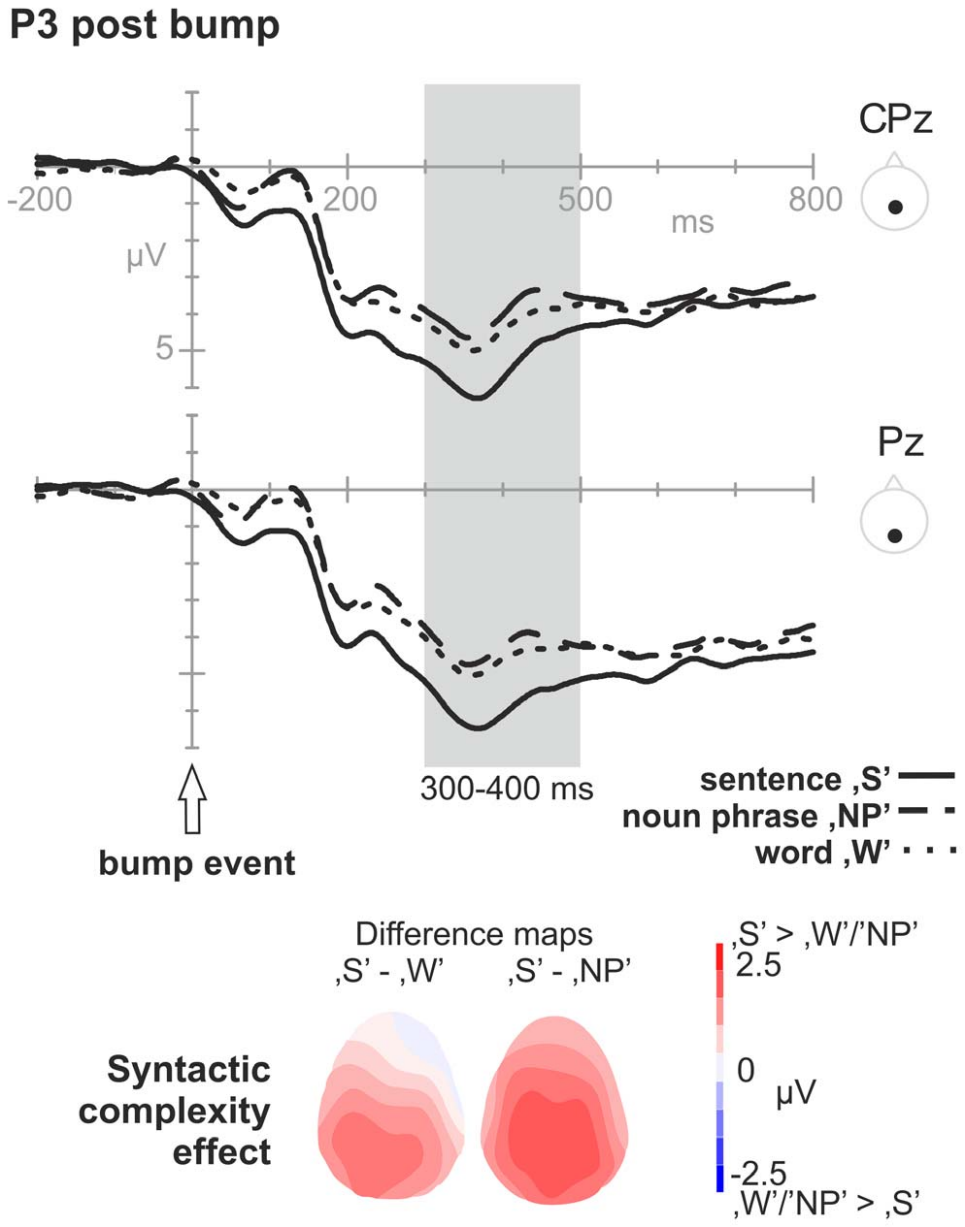
Syntactic complexity effects following verb disambiguation. Grand average ERPs, separately for the three syntactic complexity formats (‘S’, ‘NP’, ‘W’), in the post ‘bump’ epoch. Top: Signals from the centro-parietal and parietal midline electrodes (CPz Pz). The gray-shaded area indicates the *P3 bump* syntactic complexity effect (‘S’>‘NP’/‘W’) within the time window 300–500 ms post bump event. Bottom: The P3 effect distribution as a topographic map (left: ‘S’ minus ‘W’; right: ‘S’ minus ‘NP’). Note that for plotting purposes, ERP waveforms underwent a low pass filter (5 Hz, 6 dB cut-off).

*Time window 300–500 ms – P3 post bump:* A positive component was observed in the 300–500 ms time-window, having a posterior distribution. In addition to an overall marginally significant syntactic complexity effect (*F*_1.75, 47.27_ = 3.29, *p* = .052), there was an interaction between syntactic complexity and anterior-posterior distribution (*F*_2.02, 54.59_ = 4.76, *p* = .012). Simple effect analyses showed that only at posterior sites (CP, P), there was a significant syntactic complexity effect (*F*_1.76, 47.61_ = 4.45, *p* = .021) (at F, FC: *F*_1.80, 48.49_ = 1,923, *p* = .161). Pair-wise comparisons showed that ‘S’ significantly differed from ‘NP’ and differed marginally from ‘W’ (*p-corrected* = .004 and *p-corrected* = .067, respectively), where ‘S’ was more positive than ‘W’ and ‘NP’ (see Figure 5).

## Discussion

The aim of the present study was to investigate when syntactic encoding takes place during sentence planning. The sentence planning was triggered by a visual scene of moving objects. We asked participants to overtly describe these scenes using naming formats with parametrically varying syntactic complexity (using single words ‘W’, noun phrases ‘NP’, or a complete sentence ‘S’). We assumed that any variation in neural activity related to syntactic complexity would be reflected at the level of the ERP signal. Further, the design of the paradigm allowed us to temporally separate initial noun phrase planning starting at scene onset, from planning at sentence-level (occurring after all information is available, or after target action/verb disambiguation). Based on serial syntactic processing views, we expected ERP modulation around 300–500 ms after scene onset (associated with noun phrase-level syntactic planning). We based this hypothesis on previous behavioural studies on single word productions [48] and on more recent electrophysiological studies using sentence production paradigms [38], [39]. However, we did not exclude the possibility of early and non-additive neural modulations, as might be predicted by connectionist or interactive models in which visual, conceptual and syntactic processes are initiated in parallel and influence each other (e.g., [2], [14]), or might not even be viewed independently (e.g., [12]).

Behaviourally, both the number of errors and corrections showed a linear relation with syntactic complexity (the more syntax, the more errors and corrections), indicating that the intended complexity manipulation was successful. The linearity of the pattern can be interpreted as support for increasing syntactic complexity. Syntactic complexity did not influence the total speech time (TST). The TST, however, differed across action verbs, where ‘to bump into’ trials resulted in longer TSTs in all conditions, compared to ‘to fly towards’ trials. As we analyzed only the ‘to bump into’ trials in the post bump time window, this does not pose any difficulties for our results in this window. The type of action verb did not affect any of the other behavioural measures, excluding the possibility of confounding effects in the post scene epoch where both action verbs were analyzed together. With respect to the voice onset time (VOT), we found that the word-‘W’ condition differs from both the ‘NP’ and ‘S’ conditions. In particular, VOT was shorter for the ‘W’ condition (on average 1.30 s in contrast to 1.43 s, for both ‘NP’ and ‘S’), suggesting that prior to the initiation of the utterance there is already syntactic planning at the level of the noun phrase. Consistent with that, ‘NP’ and ‘S’ both require planning of noun phrases, while ‘W’ does not. The encoding requirements in ‘NP’ and ‘S’ do not differ at this moment, while they both differ from ‘W’. From this data and design, however, we cannot distinguish whether this planning entails syntactic retrieval and morpho-syntactic processing (inflections) or syntactic structure building (assuming the structure is build incrementally), or both. Either explanation would fit the observed modulation. Nevertheless, the data confirm the idea that a speaker plans in advance [4], [18], [19], in this case including already noun phrase related syntactic encoding. This is in line with previous work on the production of noun phrases [49], [50]. It should be noted, however, that both the extent and the nature of the advance planning might have been imposed by the design, because it poses constraints on the available information at this time. Previous studies have shown that the extensiveness of utterance planning can be varied depending on the speakers’ experience (in this case repetition of utterances), the circumstances (in this case availability of information), and cognitive abilities (see e.g., [18], [22]).

Compared to previous work on production of multi-word utterances, the VOTs are relatively long. In a Dutch noun phrase production study, VOTs varied around 580–670 ms, depending on the condition (picture word interference paradigm) [50]. In another study in which participants produced noun phrases, VOTs were around 660–720 ms [49]. However, an important difference between the design used in those experiments and in the present one, is the stimulation. In the current study, participants were instructed to describe a moving scene. The intended message, therefore, has to be derived from a scene consisting of several frames. In animations, as opposed to static pictures, it is not immediately clear from the start position which figures are going to be involved in the action. The information thus has to be integrated over time, which can explain the elongation of VOTs. In addition, it is important to note that the VOTs we observed are quite similar to the reaction times reported by Indefrey et al. [24], which used similar stimulation (1.29 s for ‘S’, 1.28 for ‘NP’ and 1.23 for ‘W’; reflecting similar VOTs and effects).

The observed ERPs related to overt speech production had a similar morphology for all conditions (‘W’,’NP’ and ‘S’), with a clearly visible P1, N1, P2, P3 complex and a fronto-central negativity following scene onset. Another P1, N1, P2 and P3 morphology were found after the 'bump' event (when all information including the verb was available). The first divergence across syntactic complexity conditions started approximately from 400 ms post scene onset on. We will discuss the components and their potential syntactic complexity modulations in chronological order, starting from the moment of scene onset.

### Initial Syntactic Planning

*Early components:* The first components after scene onset - P1 and N1/P2 - showed no variations across syntactic complexity conditions, indicating similar demands on the early processing functions. The early components have been associated with early perceptual processes, with attention (P1 and N1, [41]), with the early (pre)verbal stages of conceptual knowledge activation (linked to the P1, [42], [43]) and with lexical access (P2 related, [51], [52], [53]). Although it can never be ruled out completely that the response instructions resulted in differential preparatory states perceptual or conceptual processing (see e.g., [54]), the finding that these early ERP components were not modulated by our manipulation, indicates that we succeeded to keep the variance of visual and conceptual processing to a minimum over the three utterance conditions.

*P3 scene*: In a specific window, between 350 and 550 ms post scene onset, a P3-like component was clearly visible. It comprised two subcomponents: one with a posterior scalp distribution and one with a more anterior focus. No variation with syntactic complexity was found in the posteriorly distributed activity (350–450 ms post scene onset). At anterior sites variation with syntactic complexity was present within the P3 time window (450–550 ms). The word-‘W’ condition significantly differed from the noun phrase-‘NP’ and (marginally) from the sentence-‘S’ conditions, where ‘NP’ and ‘S’ elicited a higher positivity compared to ‘W’. At this time point, the action format of the scene (verb) was still ambiguous, but visual input was sufficient to give way to first noun phrase planning (nouns and adjectives). ‘W’ did not require retrieval of syntactic information or inflections at a noun phrase level, while the noun phrase ‘NP’ and sentence ‘S’ condition did (e.g., the retrieval of the syntactic gender reflected in the adjective [in Dutch *‘* ***de/het*** *’*] and the inflection of the adjectives [‘*groen>groene*’; green]). The noun phrase-related syntactic processing might in turn be reflected in higher P3 amplitudes at frontal sites.

Syntax-first language accounts assume that utterance structure is build prior to any lemma retrieval and morpho-syntactic processing [5]. The observed data would also support such a view. Either this component reflects incremental structure building of the noun phrases, or it might be that the structure is already be available, and the modulation reflects online filling of information into the structure. Processing requirements for ‘NP’ and ‘S’ do not differ for both scenario’s, but they both differ from ‘W’. Most importantly, the observed ERP modulation between 450–550 ms post scene onset indicates that this time window is sensitive to syntactic noun phrase planning.

In a previous electrophysiological production study, the P3 has been associated with conceptual and/or syntactic complexity (350–500 ms post stimulus onset, [39]), but the distribution of this component was centro-parietal (while in the current study, the effect was anterior). It seems unlikely, however, that the present effect reflects conceptual planning, as previous studies found conceptual effects in earlier time windows (e.g., 120 ms post stimulus presentation, [42]), and we did not find such early modulations. Also, the design of the current study minimized conceptual processing. Further, with respect to timing, the result is in line with studies on the time course of single word production of Indefrey and Levelt [48], with Koester and Schiller’s study on morphological encoding (priming effects were found 350–650 ms after picture onset [55]) and with Sahin et al.’s study [38] who suggested that syntactic encoding starts around 320 ms post stimulus onset (although paradigms differ). In these previous studies, syntactic structure building was not required, suggesting that the P3 effect observed in the current study does not reflect structure building only. In more general, non-linguistic terms, the P3 has been associated to a monitoring function, context updating and working memory actions (see e.g., [56]). It has also been proposed that the P3 amplitude reflects activities in a network controlled by joint operations of both attention and working memory [57]. Whether the ERP effect observed in this study reflects directly the differential demands on syntactic or differential demands on attention and working memory processes accompanying the linguistic processes cannot be disentangled. The present P3 result shows that syntactic modulation either directly (direct modulation of the P3) or indirectly (P3 modulation via attention and processing load) correlates with neural activation in this time window, indicating active syntactic processing in this time range.

*Late negativity*: The data also revealed a clear linear relation of syntactic complexity across naming conditions within a (bilateral) fronto-central negativity at 600–900 ms post scene onset. In particular, we observed that - in terms of amplitudes - ‘S’ elicited the most negative activity, followed by ‘NP’ and ‘W’. To our knowledge there is no previous report on such an ERP modulation during overt sentence production planning. We can only speculate about its interpretation here based on the complexity manipulation in our experiment. This fronto-central negativity might reflect directly (continued) syntactic structure building of the sentence to be uttered, as the syntactic structures varied across the three conditions, and can be anticipated on. Some language accounts, however, suggest that structure building already occurs relatively early in sentence production [22], which would not be in agreement with the observed rather late ERP modulation. Alternatively, it might reflect modulated working memory demands or a check/control monitoring on the appropriateness of the planning so far.

### Sentence-level Planning

*Early post bump components:* After a period of activity around baseline, without any clear distinguishable ERP components (from approximately 1000–1400 ms post scene onset), another temporal event occurred in the visual stimulation: the ‘bump’ event. At that moment in time, it became definite which of the verbs had to be used (‘to bump into’ or ‘to fly towards’). In the ‘bump’ trials - time-locked to the clearly defined 'bump' event - another set of ERP components arose that were absent in the ‘to fly towards’ trials. This absence was most likely due to the lack of a clear temporal event in the latter condition. We assume that similar cognitive processes occur in these ‘to fly towards’ trials, but they do not occur in temporal synchrony to an external event - as there is no such event. Hence, they cannot be detected by the averaging model of ERPs. The ERPs related to the ‘bump’ event showed a centrally distributed P1/N1 and a subsequent P2 complex (comprised of a posterior and more fronto-central component), but no syntactic complexity effects in these component. Analogous to the early post scene onset ERP components, these ERP components are most likely associated with more perceptual, conceptual, and basic attention processes related to the ‘bump’ event. Their insensitivity to the complexity modulation suggests again comparable visual and conceptual processing across conditions. Note that around the time of this post ‘bump’ epoch, the voice onset started on average (1.3 – 1.4 s after stimulus onset), which has been reported in the past to cause high frequency artefacts in the ERP signal (see also [30]). To avoid noise in the data caused by artefacts, we used ICA to clean the data. Independent components related to eye and muscle artefacts were filtered out of the data. As a result, we were able to observe a clear ERP morphology. Note that this is of interest from a methodological point of view, as the applied pre-processing revealed interpretable production ERPs within overt naming trials, even in relatively late time windows.

*P3 post bump:* We again observed variation with syntactic complexity within the P3-time window, but with a different, more parietal distribution (instead of a frontal distribution). In addition to differences in topographic distribution, we observed a difference in amplitude modulations across conditions. The ‘S’ condition was significantly more positive compared to ‘NP’ and marginally compared to ‘W’ (‘NP’ and ‘W’ did not differ from each other). The pattern of the complexity effect thus differed from the post scene P3 (where ‘S’ and ‘NP’ were more positive compared to ‘W’), which is an interesting functional segregation of two types of P3 effects. The difference in topography further suggests two different sources for the post scene onset P3 and post bump onset P3. At this moment in time (300–500 ms post bump event; or 1820–2020 ms post scene onset), all information was available to the participant (including the type of verb). Under the assumption that planning of the first noun phrases was already initiated immediately after scene onset, it is likely that planning within this later time window was related to local encoding of the newly available element - the verb - (e.g., lexical access, inflection) and to the (potentially continued) assembly of the utterance in general. The specific pattern of syntactic complexity effects is consistent with the idea that only in ‘S’ inflection of the verb plus assembly of all elements into a syntactically well-formed utterance was needed, while in ‘NP’ and ‘W’ this was not necessary. In the latter cases, the verb was expressed as its unmarked form (infinitive) and the word order was according to a predefined format.

Taken together, the complexity modulation in this study was reflected in both modulations in behaviour and in the ERP. In the fronto-central negativity we observed a linear pattern (the more syntactic planning, the higher the amplitude). In both the P3 following scene onset and following verb disambiguation, we observed a different complexity variation. This variation was segregated in terms of function and topography (amplitude modulations and distribution differed, respectively), indicating different neural sources. The pattern in the components suggests that the frontal P3 reflects early noun phrase planning, while the later parietal P3 indicates noun phrase assembly and integration processes. While other studies have already observed syntactic modulations in the (first) P3 time window (e.g., [38], [39]), the current study is the first to delineate syntactic sentence planning over time and to investigate the entire time window, using a more realistic display of moving objects instead of static line drawings of scenes. Therewith, it extends the findings of previous studies and demonstrates the possibility of investigating relatively late components of sentence production in a naturalistic manner.

The observation of clear, distinguishable - relatively late - time windows sensitive to syntactic encoding and a lack of any early ERP effects, seems not to support integrative accounts that assume early initiation of all processes. However, it cannot be excluded that the observed stage-like behaviour in the data emerged as a property of parallel accounts [2]. Speculatively, the data do not seem to directly support evidence for language as an emergent property, as one might expect enhanced planning for the new, un-learned utterance structures (‘W’, and in lesser extent ‘NP’) compared to the natural and learned structure of the sentences in ‘S’. However, other experimental setups would be needed to test such models directly and explicitly.

The results are in agreement with incremental encoding of the utterance, unfolding over time. From the current design, however, we cannot distinguish whether lemma retrieval/assignment and morpho-syntactic processing comes first, or only after syntactic structure building.

Although linear effects were expected, based on the parametric variation across conditions and the results of the PET study [24], [25], this was not always the case. The effect in both P3 components was not linear, but reflected a different modulation (‘W’ versus ‘NP’/‘S’, and ‘W’/’NP’ versus ‘S’, respectively). Notably, the current ERP study was able to delineate the time-insensitive PET result over time. By exploiting the high temporal resolution and certain aspects of the design, the current ERP results give us more insight in the temporal aspect of syntactic encoding. The observed ERP pattern further suggests that the overall PET effect is a summation of neural activity within different time windows and with distributed neural sources. The functional role of the LIFG and the observed linear correlation with syntactic complexity has to be re-evaluated in future experiments.

Inherent to the study of syntactic encoding is that it is impossible to create a pure manipulation of syntactic planning, as it never occurs in isolation and is difficult to manipulate without changing any of the other processes [30]. For instance, we cannot exclude differential perceptual effects across conditions caused by the instructions, as certain naming format in a given block may alter the perception and degree of attention to certain objects. The observed lack of effects in the early ERP components support the idea that any perceptual and attentional differences were negligible in the present design.

In addition, all three conditions required temporal ordering of the words into an utterance. This temporal ordering is related to conceptual encoding [15] and might involve some form of structure building. Next to the need of listing adjectives and nouns in a serial order, the infinite verb in ‘W’ and ‘NP’ is also a phrase (*“naar toe vliegen”* or “to fly towards”). Overall, ordering and minimal structure encoding might have decreased the net difference between complexity conditions. However, the lack of early ERP effects and the later, observed complexity effects, indicate that the conceptual ordering was not different across conditions, and that the complexity manipulation was sufficient to be reflected in the data, respectively.

Another limitation of the study was that the blocked design resulted in repetitions of the same response condition, and thus the same type and structure. Repeating the same structure across trials and conditions was chosen to keep conceptual processes as constant as possible, but may have potentially caused priming effects. It is plausible that structural priming effects might have occurred in the current study, facilitating the processing of a subsequent utterance with the same structure [58]. The priming might have reduced planning of the structure across trials. Nevertheless, even in face of potential structural priming effects, the syntactic complexity modulation was robustly found in several components. In future research, the use of filler trials requiring different utterance structures could be considered, to avoid structural priming effects and potentially increase the magnitude of the effects.

## Conclusion

In this study, we have examined the temporal aspects of syntactic encoding in sentence production. ERPs were cleaned from (muscle) artefacts using ICA, and we observed a clear ERP morphology. Event related potentials associated to immediate noun phrase-planning were found starting from scene onset on. By exploiting the fact that verb availability was not immediate, but temporally defined by an event, we were able to investigate relatively late ERPs related to noun phrase assemblies and overall sentence integration. More specifically, we found that overt description of a movie-like scene elicited very similar P1/N1/P2 components across all complexity conditions (words, noun phrases, or sentence format). From 400 ms onwards, conditions started to deviate in specific time windows. In particular, we found three components showing a modulation with syntactic complexity: following scene onset an anterior P3 scene effect (at 450–550 ms post scene onset; ‘S’/‘NP’>‘W’) and a fronto-central negativity (at 600–900 ms post scene onset; ‘S’>‘NP’>‘W’) were observed, and following the ‘bump’ event another, more posterior, P3 effect (300–500 ms after verb availability; ‘S’>‘NP’/‘W’). We interpret the components in the first time window - the *P3 scene* and *fronto-central negativity* - as related to syntactic encoding of noun phrases. The P3 related syntactic encoding here seems to involve the retrieval of syntactic information, such as inflections, and the assembly of words into phrases, in which ‘S’/‘NP’ differ from ‘W’. The late negativity seems sensitive to syntactic structure building as it modulates differently across the three conditions. The ERP component in the later time window - the *P3 bump* - is related to more global syntactic planning at the sentence level. This may involve encoding of the verb and continued assembly of the utterance, in which ‘S’ differs from ‘NP’/‘W’. The data show that the P300 time window is sensitive to syntactic planning, both at noun phrase-level and at sentence-level. The functional segregation and differential topographical distributions of the P3 components further indicates different neural sources, suggesting that noun phrase planning and sentence-level planning require different cognitive operations.
